# OPN Polymorphism Is Related to the Chemotherapy Response and Prognosis in Advanced NSCLC

**DOI:** 10.1155/2014/846142

**Published:** 2014-08-05

**Authors:** Yanzhang Hao, Jianwei Liu, Ping Wang, Feng Wang, Zeshun Yu, Mianli Li, Shaoshui Chen, Fangling Ning

**Affiliations:** ^1^Department of Oncology, Affiliated Hospital of Binzhou Medical University, China; ^2^Department of Chest Surgery, Affiliated Hospital of Binzhou Medical University, China; ^3^Department of Infectious Diseases, Affiliated Hospital of Binzhou Medical University, China

## Abstract

*Background.* Osteopontin (OPN) is associated with prognosis of patients with non-small-cell lung cancer (NSCLC). However, little is known about the association between OPN gene polymorphism and the chemotherapy response in NSCLC patients. *Methods.* A total of 497 patients with inoperable advanced stage of NSCLC (stages III B and IV NSCLC) were enrolled. All patients had received platinum-based chemotherapy. OPN gene polymorphisms at 156 GG/G, 443 C/T, and −66T/G were determined. *Results.* The genotypes and allele frequency of −443C>T were significantly different between the responders and nonresponders. Responders had a markedly higher frequency of −443TT genotype than responders (40.71% versus 19.09%, *P* < 0.001). With CC as reference, the TT genotype carriers had a higher chance to be well responders (adjusted OR = 4.43, 95% CI: 2.60–7.53, adjusted *P* < 0.001). The median overall survival time for patients with −443CC, −443CT, and −443TT genotype carriers was significantly different. Multivariate Cox proportional hazards regression models showed that OPN −443C>T gene polymorphisms were closely correlated to poor NSCLC prognosis. *Conclusion.* OPN −443C>T gene polymorphism may be used as a molecular marker to predict the treatment response to chemotherapy in advanced NSCLC patients.

## 1. Introduction

Non-small-cell lung cancer (NSCLC) accounts for approximately 80–85% of all lung cancer [1]. The prognosis of NSCLC is poor and it is estimated that the 5-year survival rate for NSCLC is less than 15% [2]. Many NSCLC patients are diagnosed at late stage, missing the chance for surgical resection. For these patients, platinum-based chemotherapy is presently the standard first-line chemotherapy [3]. However, the response rate to platinum-based regimen of advanced NSCLC was less than 30%. Epidemiological studies reveal the genetic factor, for example, gene polymorphism of certain genes, contributing to the different chemotherapy response in NSCLC patients [4, 5]. However, the efficacy and reliability of these candidate remain to be further determined.

Osteopontin (OPN) is a sibling glycoprotein that was first identified in osteoblasts [6]. Osteopontin (OPN) is a multifunctional cytokine involved in cell survival, migration, and adhesion which is associated with tumorigenesis, progression, and metastasis. OPN is also implicated in tumorigenesis and has been proposed as a cancer marker [7–11]. OPN is also expressed in lung tissue [12]. Clinical study showed that high OPN expression is associated with poor survival of patients with stage I non-small-cell lung cancer, suggesting that OPN could be a candidate target for cancer therapy [13, 14]. OPN is a prognostic marker in curatively resected NSCLC [15]. Overexpression of OPN is associated with more aggressive phenotypes in human non-small-cell lung cancer [16].

Several single nucleotide polymorphisms (SNPs) in the human OPN encoding gene have been identified. Several polymorphisms in the human OPN encoding gene have been identified in different populations, of which the −156GG/G, −443C/T, and −66T/G polymorphisms were mostly studied. These OPN genetic polymorphisms that have been reported are associated with inflammatory diseases.

Based on the increased expression of OPN in the NSCLC tissue, we postulate that the OPN gene polymorphism is related to the chemotherapy response in NSCLC patients. In this study, we enrolled inoperable NSCLC (stages IIIB and IV) receiving platinum-based chemotherapy to test this hypothesis.

## 2. Methods

### 2.1. Patient Enrollment

A total of 497 patients with inoperable advanced stage of NSCLC, namely, stages III B and IV NSCLC, confirmed cytologically or histologically were enrolled into this study. To avoid the potential influence of poor clinical conditions on chemotherapy response, other eligibility criteria included normal blood chemistries (hemoglobin >9 g/dL, neutrophil count >1500/mm^3^, and platelet count >100 000/mm^3^), hepatic function (bilirubin <1.5 times the normal upper limit and aspartate aminotransferase and alanine aminotransferase <2 times the normal upper limit) and renal function (creatine clearance rate >50 mL/s), and normal electrocardiogram at the beginning of treatment. The study was approved by the ethics committees of our hospital and written informed consent was obtained from each participant.

### 2.2. Chemotherapy Regimens and Therapeutic Effect Evaluation

All patients had received platinum-based chemotherapy after diagnosis. Patient responses to treatment were determined by the World Health Organization criteria [17], which classify the response into four categories: complete response (CR), partial response (PR), stable disease (SD), and progressive disease (PD). CR and PR were combined as well responders, and SD and PD were grouped as poor responders. The chemotherapy response was assessed by two independent oncologists who were blind to our study. Overall survival (OS) and progression free survival (PFS) were the end points in this study. OS was calculated from the date of chemotherapy to the date of last follow-up or death from any cause. PFS was defined as the interval between the date of chemotherapy and the date of confirmed relapse.

### 2.3. OPN Gene Polymorphisms

DNA was extracted from peripheral whole blood using a Qiagen DNA Isolation Kit (Qiagen, Valencia, CA, USA). The single nucleotide polymorphisms on the promoter region of OPN gene were determined using TaqMan 5′ allelic discrimination assay. It was performed using a commercially available kit Assays-on-DemandTM SNP genotyping products (Applied Biosystems, Foster City, CA). SNP amplification assays were used according to the manufacturer's instructions. In short, 10 ng of sample DNA in 25 *μ*L of reaction solution contains 12.5 *μ*L of the 2× TaqMan Universal PCR Mix (Applied Biosystems), and 1.25 *μ*L of predeveloped assay reagent from the SNP genotyping product contains two primers and two MCB-Taqman probes. Reaction condition consisted of preincubation at 50°C for 2 min, at 95°C for 10 min, and followed by 40 cycles of 95°C for 15 s and 60°C for 1 min. Amplifications were performed in an ABI Prism 7500 Sequence Detection System (Applied Biosystems).

### 2.4. Statistical Analysis

To estimate the deviation of frequency of gene alleles in tested population, we performed the Hardy-Weinberg equilibrium using *χ* ^2^ tests. Multivariate logistic regression analysis was used to determine the influence of OPN polymorphisms on the chemotherapy response, controlling potential confounding conventional risk factors. A forward stepwise (likelihood ratio) procedure was used for multivariable analysis. The Kaplan-Meier method was used to calculate OS. The differences in OS were compared using log-rank test. Data were analyzed with the SAS 9.2 package (SAS INC. NC. USA). The results were considered statistically significant at *P* < 0.05 using a 2-tailed test.

## 3. Results

Among all patients that received platinum-based chemotherapy, 311 were chemotherapy responder (CR + PR) and 430 were nonresponder (SD + PD). Nonresponders had higher prevalence of smokers, patients with stage IV and with poor differentiation than responders (all *P* < 0.05, Table 1). There were no significant differences in sex, age, histology, and chemotherapy regimens between responders and nonresponders (all *P* > 0.05).

Genotype frequencies of OPN gene polymorphisms in chemotherapy responder and nonresponders were found to be fit in the Hardy-Weinberg equilibrium (all *P* > 0.05).  Table 2 shows the genotypes and allele frequency of −443C>T was significantly different between the responders and nonresponders. Responders had a markedly higher frequency of −443TT genotype than responders (40.71% versus 19.09%, *P* < 0.001). With CC as reference, multivariate logistic regression analysis showed that the TT genotype carriers had a higher chance to be well responders (adjusted OR = 4.43, 95% CI: 2.60–7.53, adjusted *P* < 0.001) with adjustment for age, sex, smoke status, histology, cancer stage, and chemotherapy regimens. The CT genotype carriers are also associated with chemotherapy response (adjusted OR = 1.94, 95% CI: 1.18–3.10, adjusted *P* = 0.008). The T allele carriage represented a higher possibility to be chemotherapy responders after adjustment with the above mentioned clinical variables (adjusted OR = 1.68, 95% CI: 1.30–2.16, *P* < 0.001) compared with C allele carriage. The genotypes and allele frequency of −156/G>GG and −66T>G were not significantly different between well responders and poor responders (all *P* > 0.05).

**Figure 1:**
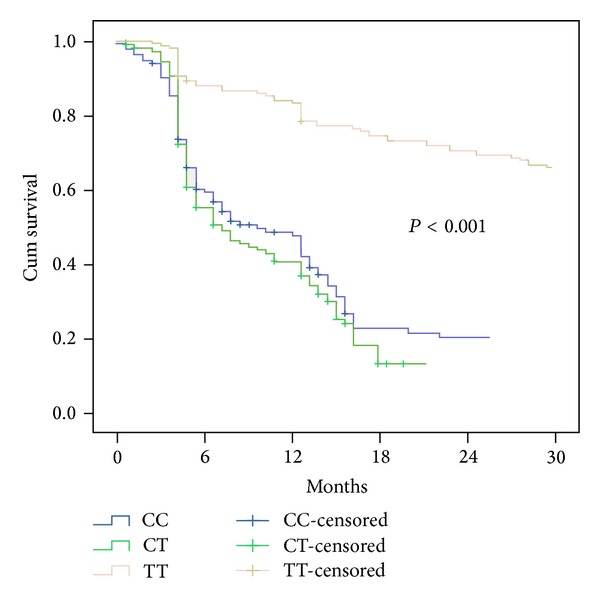
The Kaplan-Meier curves for the effect of −443CC, −443CT, and −443TT genotype on OS in NSCLC patients.

The associations between OPN genotype and OS of NSCLC patients were studied by log-rank test. There were no significant differences in OS based on the OPN genotypes at −156/G>GG and −66T>G locus (all Log-rank *P* > 0.05, data not shown). In contrast, the median OS time for patients with −443CC, −443CT, and −443TT genotype carriers was significantly different (Table 3). The Kaplan-Meier curves for the effect of −443CC, −443CT, and −443TT genotype on OS are shown in Figure 1.

Next, the multivariate Cox proportional hazards regression models were used to estimate the hazard ratios (HR) for OS, with adjustment for age, sex, smoking status, histology, stage, and response status.  Table 4 shows that the tumor differentiation (well + moderate versus poor), chemotherapy response (well response versus poor response), and −443C>T gene polymorphism (CC versus TT) were closely correlated to poor NSCLC prognosis.

## 4. Discussion

It is very important to select the patients who response to chemotherapy to achieve better clinical outcome and to avoid the side effect of chemotherapy. In this study we reported that OPN gene polymorphisms influence the treatment response and clinical outcomes of advanced NSCLC patients receiving platinum-based chemotherapy. We found that the −443C>T polymorphisms significantly associated with the chemotherapy response and predict prognosis of NSCLC patient receiving chemotherapy. The TT genotype carriers had a markedly higher chance to be responders to chemotherapy. The −443TT genotype carriers tend to have better prognosis of NSCLC. To the best of our knowledge, this is the first study regarding the OPN genetic polymorphism and treatment response in NSCLC.

OPN overexpressed in the NSCLC tumor tissues has been reported significantly correlated with TNM stages and lymph metastasis [18]. There are several polymorphic sites in the regulatory element of the OPN promoter, and SNPs at nucleotide −443 are frequently detected and reported involved in regulation of OPN expression in normal cells [18–20]. A recent study on melanoma metastases found that those homozygous for the −443C allele expressed significantly higher levels of OPN mRNA compared to those that were either heterozygous (CT) or homozygous for the −443 T allele [21]. The OPN promoter polymorphisms at locus −443 significantly affect the metastasis and prognosis of human hepatocellular carcinoma [19]. The functional −443T/C osteopontin promoter polymorphism influences osteopontin gene expression in melanoma cells via binding of c-Myb transcription factor [21]. OPN −443C>T genetic polymorphism and tumor OPN expression are associated with the risk and clinical features of papillary thyroid cancer in a Chinese cohort [22, 23].

A previous study examined the possibility that SNP in the promoter region of the OPN at nt −443 is a marker predicting the therapeutic efficacy of pegylated interferon- (peg-IFN-alpha2b-) ribavirin combination therapy in Egyptian patients with chronic hepatitis C [24]. Serum OPN protein level and T/T homozygotes of SNP at −443 were significant predictors for response. SNP in the promoter region of OPN at nt −443 and serum OPN protein level are predictors of response to the efficacy of peg-IFN-alpha2b-ribavirin therapy in patients with chronic hepatitis C [25, 26]. Another study reported that SNPs in the promoter region of OPN may be useful as a marker to predict the efficacy of IFN-based therapies in patients with chronic hepatitis C [27, 28]. OPN −156G/GG did not affect the treatment response to anti-TNF therapy in patients with rheumatoid arthritis [29]. In this study, we reported that the OPN gene polymorphism at −443C>T is also associated with the chemotherapy in NSCLC patients.

The prognostic role of OPN gene polymorphism in cancer patients was reported as well. A recent study showed that the survival rates for patients with the −443CC genotype were significantly lower than the survival rates of the other two genotypes (−443CT and −443TT) [30, 31]. In that study, the −443C/T polymorphism is also correlated with bone metastasis significantly. These data suggest that OPN gene polymorphism is a potential predictive marker of survival in lung cancer patients. Our data are consistent with this observation. We found that the −443C>T polymorphisms significantly associated with the chemotherapy response and predict prognosis of NSCLC patient receiving chemotherapy. The TT genotype carriers had a markedly higher chance to be responders to chemotherapy. The −443TT genotype carriers tend to have better prognosis of NSCLC.

There are limitations in the present study; one of them is because all of the subjects are Chinese individuals, and the results should be interpreted with caution and need to be confirmed in larger and ethnically divergent population samples. On the other hand, the number of patients in the current study is relatively small, so the large-population research is needed to make stronger conclusion about the association between bone metastasis formation and −433 polymorphisms.

## Figures and Tables

**Table 1:** Patient characteristics between well responder and poor responders to platinum-based chemotherapy.

Characteristics patient	Well responder		Poor responder		*P*
Age (years)	52.8 ± 3.2	%	53.0 ± 5.1	%	0.083
Gender					
Male	100	59.88%	198	60.00%	0.281
Female	67	40.12%	132	40.00%	
Smoke status					
nonsmokers	110	65.87%	163	49.39%	0.025
smoker	57	34.13%	167	50.61%	
Histology					
Squamous cell carcinoma	80	47.90%	166	50.30%	0.225
Adenocarcinoma	87	52.10%	164	49.70%	
Stage					
IIIB	101	60.48%	165	50.00%	
IV	66	39.52%	165	50.00%	
Differentiation					
Well	68	40.72%	87	26.36%	0.016
Moderate	54	32.34%	135	40.91%	
Poor	45	26.95%	108	32.73%	
Chemotherapy regimens					
DDP/CBP + TAX/TXT/DOC	87	52.10%	110	33.33%	0.15
DDP/CBP + GEM	65	38.92%	122	36.97%	
DDP/CBP + NVB	15	8.98%	98	29.70%	

**Table 2:** The genotype frequencies for HIF-1*α* polymorphisms between well responders and poor responders to platinum-based chemotherapy.

OPN genotype	Well responders *N* = 167	%	Poor responders *N* = 330	%	adjusted OR	95% CI		Adjusted *P*
	*N*	%	*N*	%				

−443C>T								
CC	29	17.37%	119	36.06%	1.00			
CT	70	41.92%	148	44.85%	1.94	1.18	3.19	0.008
TT	68	40.72%	63	19.09%	4.43	2.60	7.53	<0.001
C	128	38.32%	543	51.03%	1.00			
T	206	61.68%	521	48.97%	1.68	1.30	2.16	<0.001
−156/G>GG								
GG	44	26.35%	89	26.97%	1.00			
GGG	80	47.90%	143	43.33%	1.13	0.72	1.78	0.593
GGGG	43	25.75%	98	29.70%	0.89	0.53	1.48	0.646
G	168	50.30%	543	51.03%	1.00			
GG	166	49.70%	521	48.97%	1.03	0.81	1.32	0.815
−66T>G								
TT	48	28.74%	98	29.70%	1.00			
TG	71	42.51%	142	43.03%	1.02	0.65	1.60	0.928
GG	48	28.74%	90	27.27%	1.09	0.67	1.78	0.734
T	167	50.00%	543	51.03%	1.00			
G	167	50.00%	521	48.97%	1.04	0.82	1.33	0.742

**Table 3:** The associations between −443T>C genotype and OS by log-rank test.

OPN polymorphisms	Median OS, mo (95% CI)	Log-rank *P*
−443C>T		
CC	11.6 (6.6–15.2)	<0.001
CT	10.1 (4.0–17.2)	
TT	19.9 (8.6–23.3)	

**Table 4:** HR for prognosis of NSCLC patients that underwent chemotherapy.

Factors	HR	95% CI	*P*
Chemotherapy response			
Well response	1		
Poor response	2.87	1.54–4.89	<0.001
Differentiation			
Well + moderate	1		
Poor	2.02	1.13–3.62	<0.001
OPN −443C>T			
TT	1		
CC	2.46	1.92–4.83	<0.001
